# Pathological changes of distal motor neurons after complete spinal cord injury

**DOI:** 10.1186/s13041-018-0422-3

**Published:** 2019-01-09

**Authors:** Kazuya Yokota, Kensuke Kubota, Kazu Kobayakawa, Takeyuki Saito, Masamitsu Hara, Ken Kijima, Takeshi Maeda, Hiroyuki Katoh, Yasuyuki Ohkawa, Yasuharu Nakashima, Seiji Okada

**Affiliations:** 1 Department of Orthopaedic Surgery, Graduate School of Medical Sciences, Kyushu University, 3-1-1 Maidashi, Higashi-ku, Fukuoka, 812-8582 Japan; 2 Department of Immunology and Neuroscience, Medical Institute of Bioregulation, Kyushu University, 3-1-1 Maidashi, Higashi-ku, Fukuoka, 812-8582 Japan; 3 Department of Orthopaedic Surgery, Spinal Injuries Center, 550-4 Igisu, Iizuka, Fukuoka 820-8508 Japan; 4 Department of Orthopaedic Surgery, Tokai University School of Medicine, 143 Shimokasuya, Isehara, Kanagawa 259-1193 Japan; 5 Department of Transcriptomics, JST-CREST, Medical Institute of Bioregulation, Kyushu University, 3-1-1 Maidashi, Higashi-ku, Fukuoka, 812-8582 Japan

**Keywords:** Spinal cord injury, Chronic phase, Laser microdissection, Cell-selective gene expression, Synaptogenic potential, Motor neurons

## Abstract

Traumatic spinal cord injury (SCI) causes serious disruption of neuronal circuits that leads to motor functional deficits. Regeneration of disrupted circuits back to their original target is necessary for the restoration of function after SCI, but the pathophysiological condition of the caudal spinal cord has not been sufficiently studied. Here we investigated the histological and biological changes in the distal part of the injured spinal cord, using a mice model of complete thoracic SCI in the chronic stage (3 months after injury). Atrophic changes were widely observed in the injured spinal cord both rostral and caudal to the lesion, but the decrease in area was mainly in the white matter in the rostral spinal cord while both the white and gray matter decreased in the caudal spinal cord. The number of the motor neurons was maintained in the chronic phase of injury, but the number of presynaptic boutons decreased in the lumbar motor neurons caudal to the lesion. Using laser microdissection, to investigate gene expressions in motor neurons caudal to the lesion, we observed a decrease in the expressions of neuronal activity markers. However, we found that the synaptogenic potential of postsynapse molecules was maintained in the motor neurons after SCI with the expression of acetylcholine-related molecules actually higher after SCI. Collectively, our results show that the potential of synaptogenesis is maintained in the motor neurons caudal to the lesion, even though presynaptic input is decreased. Although researches into SCI concentrate their effort on the lesion epicenter, our findings suggest that the area caudal to the lesion could be an original therapeutic target for the chronically injured spinal cord.

**Electronic supplementary material:**

The online version of this article (10.1186/s13041-018-0422-3) contains supplementary material, which is available to authorized users.

## Introduction

Traumatic spinal cord injury (SCI) is a severely debilitating injury with permanent motor/sensory dysfunction that imposes considerable mental and economic burdens [1] and leads to a marked reduction in the quality of life [2]. Patients with incomplete SCI experience a recovery in function that can begin immediately after injury and continue for several weeks after injury [3, 4] while the functional recovery in patients with complete SCI is severely limited. Ongoing research into the treatment for SCI has led to the development of numerous strategies to improve the impairment of incomplete SCI, but the unfortunate truth is that the treatment options for patients with complete SCI are virtually nonexistent [5, 6]. Therefore, there is a great demand for developing treatment strategies for complete SCI; but before we can treat complete SCI, we need to understand the pathophysiological condition of the spinal cord following complete SCI.

After SCI, axons and dendrites that lost connection to their original neural pathways degenerate in a process called Wallerian degeneration or axonal dieback, which starts at the site of injury and proceeds in a direction away from the injury epicenter [7, 8]. Previous reports exploring the pathological events that occur in the sites remote to the lesion in the chronic phase of SCI [9, 10] provide information that is vital when considering future strategies for establishing treatment approaches for SCI. Given that there is evidence demonstrating that the synaptic connections of lumbar motor neurons affect locomotor function even after incomplete thoracic SCI [11], it is clear that the distal neuronal components should not be considered irrelevant. Indeed, a better understanding of the synaptic pathology of the motor neurons distal to the lesion may turn out to be a critical component in the development of novel treatments to promote axonal connectivity and bring about meaningful functional recovery.

In this study, we investigated the histological and biological changes that occur distal to the lesion in a severe thoracic SCI mice model. Utilizing laser microdissection (LMD) to isolate single cells for study, we examined the synaptogenic potential of motor neurons in lumbar spinal cords (which are remote to the lesion) and demonstrated that even though the synaptic input decreases in distal motor neurons after severe SCI, the synaptogenic potential is maintained. Our findings confirm that the neural components caudal area to the lesion undergo pathological change after severe SCI, but contrary to expectations, our results demonstrate that the area caudal to the lesion could be a potential target to improve functional outcome after SCI.

## Results

### Severe SCI leads to atrophic changes of the spinal cord distal to the lesion

**Fig. 1:**
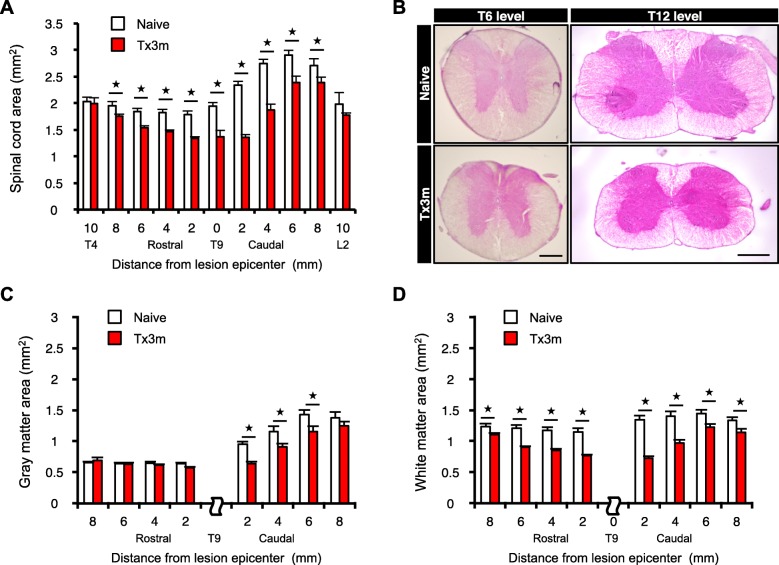
Severe SCI leads to widespread atrophic changes in the spinal cord. **a** The results of the area of spinal cords in the uninjured group (Naive group) and in the injured group at 3 month after complete SCI (Tx3m group) (*n* = 8 mice per group). **b** Hematoxylin-eosin (HE) stained axial sections of spinal cord taken rostral (T6 level) and caudal (T12 level) to the injured site (T9 level). **c** The area of gray matter in the Naive and Tx3m groups (*n* = 8 mice per group). **d** The area of white matter in the Naive and Tx3m groups (*n* = 8 mice per group). *P < 0.05, n.s. = not significant (*P* > 0.05), Wilcoxon rank sum test (**a**, **c**, and **d**). Data are presented as the mean ± SEM. Scale bars: 50 μm (**b**, left panel); 100 μm (**b**, right panel)

In accordance to the consensus that defines the chronic phase of SCI as 3 months following SCI [12, 13], we histologically examined the spinal cord tissues rostral and caudal to the lesion site at 3 months after SCI in a mouse model of severe thoracic SCI (T9 complete transection: Tx3m group) and compared it to an uninjured spinal cord (Naive group). Hematoxylin and eosin (HE) staining showed that the areas of the injured spinal cord both rostral and caudal to the lesion site were significantly decreased in axial sections compared to the uninjured spinal cord (Fig. 1a and b), suggesting that pathological changes are widely found in the rostral and caudal areas of the injured spinal cord in the chronic phase of SCI. Interestingly, whereas the area rostral to the lesion decreased mainly in the white matter, the area caudal to the lesion decreased in both the white matter and the gray matter (Fig. 1c and d).

### The descending presynaptic inputs to lumbar motor neurons decrease in the chronic phase of SCI

**Fig. 2:**
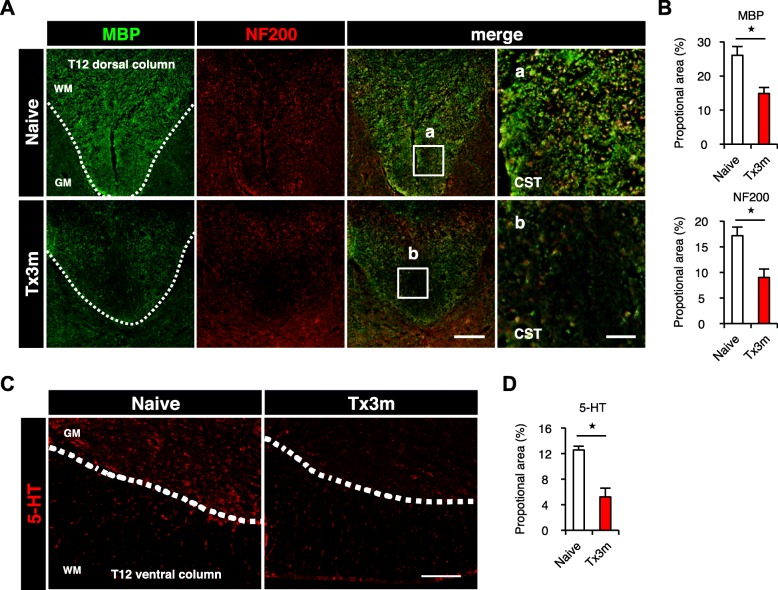
The number of neural fibers decreases in the spinal cord caudal to the lesion. **a** Immunohistochemistry for the corticospinal tract (CST) in the dorsal column of the lumbar spinal cord, stained for MBP (green) and NF200 (red). The rightmost images are magnifications of the boxed areas. **b** The ratio of MBP-positive area or NF200-positive area to transverse spinal cord area in the Naive and Tx3m groups (*n* = 5 mice per group). **c** Immunohistochemical analysis of serotonergic neural fibers in the ventral horn of the lumbar spinal cord, stained with 5-HT (red). **d** The ratio of 5-HT-positive area to transverse spinal cord area in the Naive group and in the Tx3m group (*n* = 4 mice per group). *P < 0.05, n.s. = not significant (*P* > 0.05), Wilcoxon rank sum test (**b** and **d**). Data are presented as the mean ± SEM. Scale bars: 50 μm (**a**, left three panels); 10 μm (**a**, right panels); 100 μm (**c**)

The decrease in the gray matter distal to the lesion indicates that pathological changes occur in the cellular and cytoplasmic components of this area. In order to examine the changes responsible for the decrease in the white matter, quantitative analysis of neural fibers in the distal sites in the chronic phase of SCI were conducted using immunofluorescence staining. In the dorsal region of the lumbar spinal cord [14], NF200-positive corticospinal tract fibers and MBP-positive myelin-specific proteins significantly decreased in the Tx3m group compared to the Naive group (Fig. 2a and b). In the ventral region of the lumbar spinal cord, the area of 5-HT-positive raphe spinal tracts in the Tx3m group was significantly smaller than that observed in the Naive group. (Fig. 2c and d). These results suggest that by the chronic phase of SCI, there are decreased descending axonal inputs to the lumbar motor neurons.

**Fig. 3:**
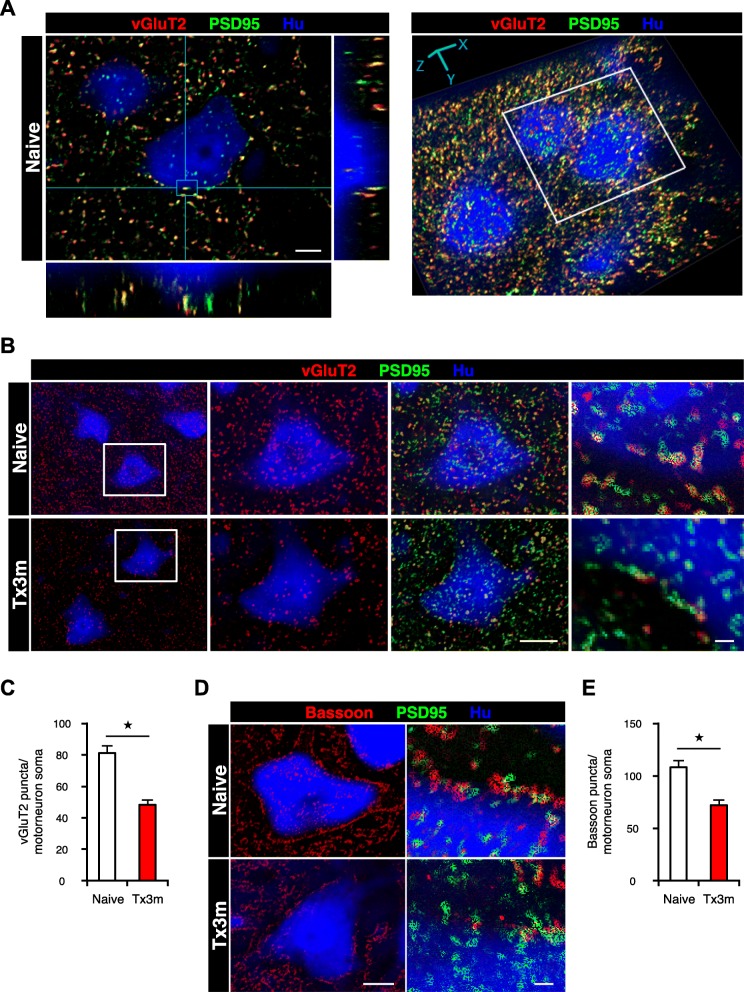
The presynaptic input to the lumbar motor neurons decreases in the chronic phase of SCI. **a** Representative images of lumbar motor neurons, showing 3D colocalization of vGluT2-positive presynaptic boutons (red) and PSD95-positive postsynaptic boutons (green). **b** Immunohistochemical analysis of excitatory synaptic boutons in lumbar motor neurons, stained for vGluT2 (red), PSD95 (green), and Hu (blue). The two middle images are magnifications of the boxed areas. **c** Quantification of the vGluT2-positive excitatory presynaptic boutons in lumbar motor neurons of the Naive and Tx3m groups (*n* = 8 neurons from 4 mice). **d** Immunohistochemistry for pan-synaptic boutons in lumbar motor neurons, stained for Bassoon (red), PSD95 (green), and Hu (blue). **e** Quantification of Bassoon-positive pan-presynaptic boutons in lumbar motor neurons of the Naive and Tx3m groups (*n* = 8 neurons from 4 mice). *P < 0.05, Wilcoxon rank sum test (**c** and **e**). Data are presented as the mean ± SEM. Scale bars: 5 μm (**a**); 10 μm (**b**, two middle panels; **d**, left panel); 1 μm (**b** and **d**, right panels)

In order to study whether the decrease in distal neural fibers leads to changes in the synaptic connectivity of lumbar motor neurons in the chronic phase of SCI, we created three-dimensional reconstruction of the lumbar spinal cord from histological images and quantified the number of synaptic boutons in the lumbar motor neurons (Fig. 3a). In particular, we focused on the motor neurons located at the 12th thoracic vertebral level, which corresponds to the lumbar enlargement at the L3 and L4 spinal cord level where the number of motor neurons is highest [15, 16]. Large (cell diameter > 20 μm) Hu-positive neurons in the ventral horn were selected as motor neurons based on morphological criteria. The identity of these Hu-positive neurons as motor neurons was confirmed with ChAT immunostaining (Additional file 1: Figure S1A). The reconstructed images confirmed that the number and size of motor neurons in the lumbar spinal cord were comparable between the Naive group and the Tx3m group (Additional file 2: Figure S2A and B). Quantification of presynaptic boutons revealed a significant decrease in both vGluT2-positive excitatory synaptic boutons (Fig. 3b and c) and Bassoon-positive pan-presynaptic boutons (Fig. 3d and e) in the Tx3m group compared to that in the Naive group, suggesting that the synaptic inputs to the lumbar motor neurons in the chronic phase of SCI in conjunction with the decreased input from the descending neural fibers.

### Cell-specific gene expression analysis reveals decreased neuronal activity in lumbar motor neurons of the chronically injured spinal cord

**Fig. 4:**
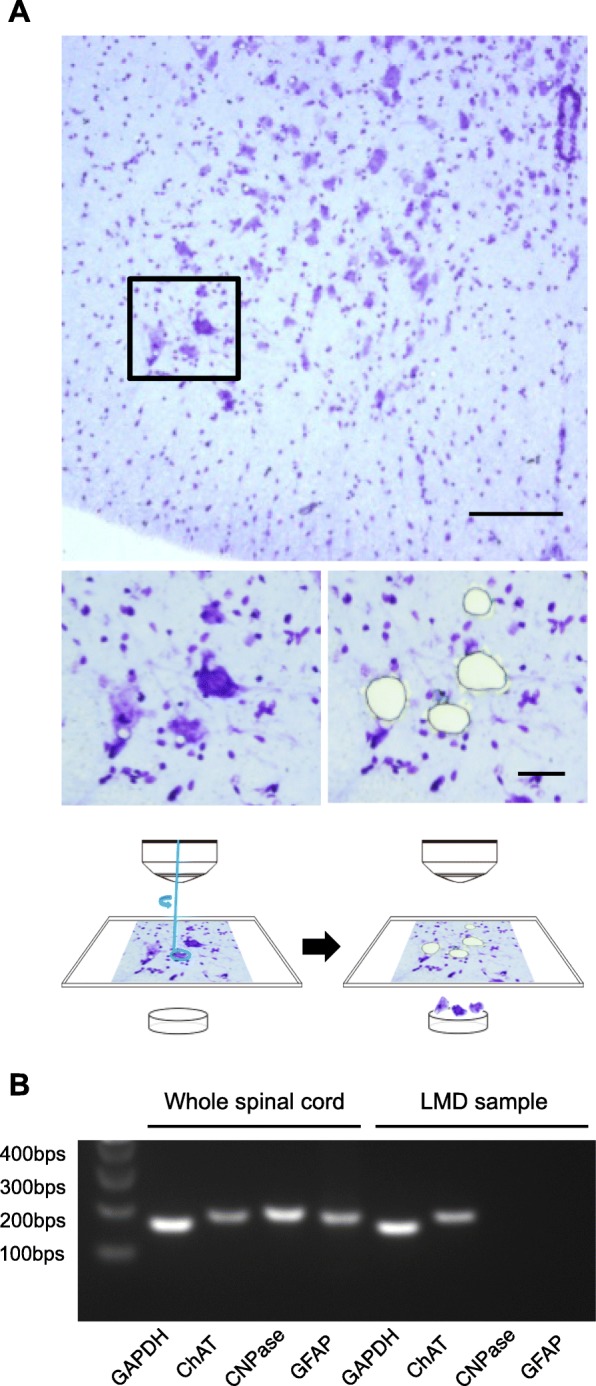
Laser microdissection (LMD) enables isolation of lumbar motor neurons. **a** Nissl-stained axial sections of the lumbar spinal cord before (lower left image) and after microdissection (lower right image) of the boxed areas in the upper image. **b** The identity of neuronal cells isolated by LMD were confirmed by their expression of the motor neuron marker ChAT and not oligodendrocyte (CNPase) or astrocyte (GFAP) markers. Scale bars: 100 μm (**a**, upper panel); 20 μm (**a**, lower panels)

**Fig. 5:**
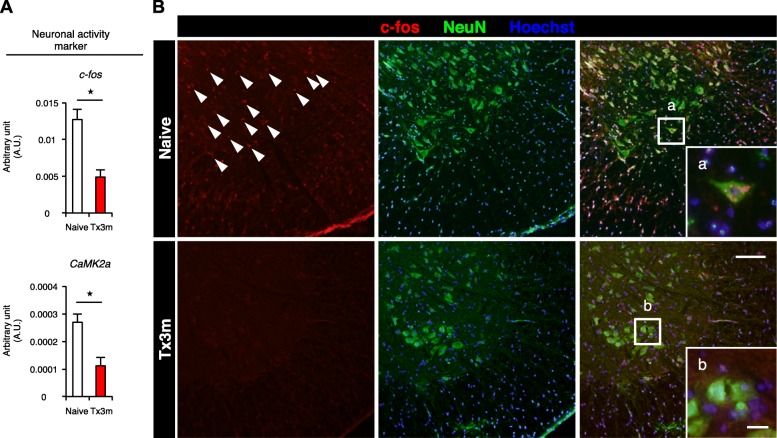
Gene expression of lumbar motor neurons reveal decreased neuronal activity in the chronic phase of SCI. **A:** The mRNA expression of neuronal activity markers c-fos and CaMK2a in lumbar motor neurons isolated by LMD from the Naive and Tx3m groups (*n* = 8 mice per group). **B:** Immunohistochemistry for the neuronal activity markers c-fos (red) and NeuN (green) in the lumbar motor neurons, with nuclear counterstain in Hoechst (blue). The lower right images are magnifications of the boxed areas. *P < 0.05, Wilcoxon rank sum test (**A**). Data are presented as the mean ± SEM. Scale bars: 100 μm (**B**); 10 μm (**B-a** and **B-b** in the boxed areas)

Having established that lumbar motor neurons have significantly decreased descending inputs in the chronic phase of SCI, we then performed laser microdissection (LMD) to specifically collect lumbar motor neurons (Fig. 4a) and examine cell-specific gene expression by quantitative RT-PCR. The selective collection of motor neurons by LMD was confirmed by the expression of ChAT in the motor neurons (Fig. 4b). The gene expression of neuronal activity markers, such as c-fos and CaMK2a [17, 18], was significantly lower in the Tx3m group compared to the Naive group (Fig. 5A), and this observation was immunohistochemically confirmed by the significant decrease in c-fos expression in the Tx3m group (Fig. 5B). However, we could not establish whether this was related to the decreased synaptic input which was observed in the lumbar motor neurons (Fig. 3).

### Lumbar motor neurons maintain expression of post-synaptic molecules in the chronic phase of SCI

**Fig. 6:**
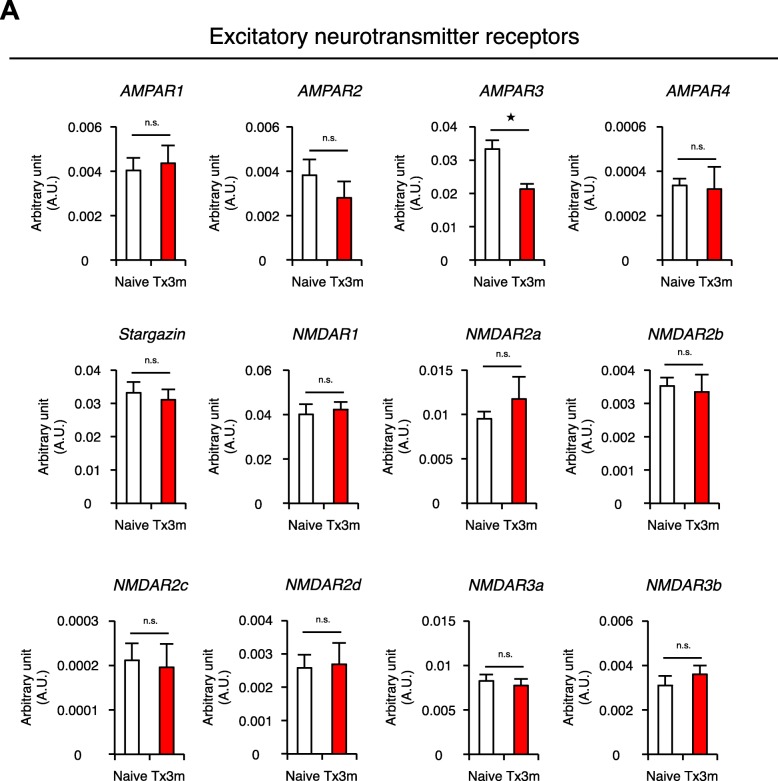
Lumbar motor neurons maintain expression of most excitatory neurotransmitter receptors in the chronic phase of SCI. **a** mRNA expression of excitatory neurotransmitter receptors in lumbar motor neurons isolated by LMD in the Naive and Tx3m groups (*n* = 8 mice per group). *P < 0.05, n.s. = not significant (*P* > 0.05), Wilcoxon rank sum test (**a**). Data are presented as the mean ± SEM

**Fig. 7:**
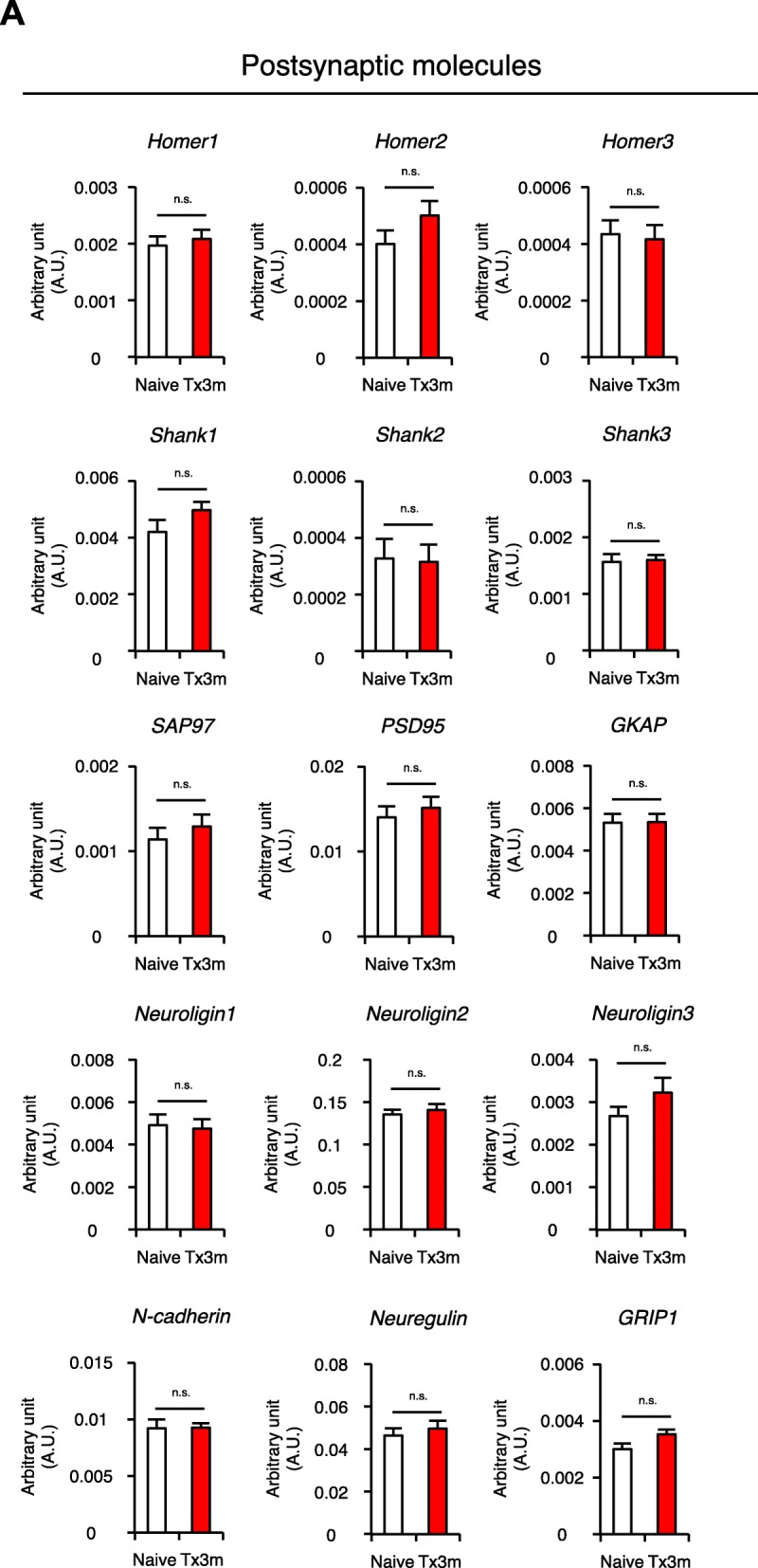
Lumbar motor neurons maintain expression of postsynaptic molecules. **a** mRNA expression of postsynaptic molecules in lumbar motor neurons isolated by LMD in the Naive and Tx3m groups (*n* = 8 mice per group). *P < 0.05, n.s. = not significant (*P* > 0.05), Wilcoxon rank sum test (**a**). Data are presented as the mean ± SEM

Postulating that the decreased neuronal activity of the lumbar motor neurons in chronic SCI is also attributed to changes in the synaptogenic potential of the neurons, we investigated the gene expression of post-synaptic molecules in motor neurons selectively collected by LMD (Fig. 4). RT-PCR revealed that the expression of postsynaptic excitatory neurotransmitter receptors AMPAR1, AMPAR2, AMPAR4, Stargazin (also known as CACNG2), NMDAR1, NMDAR2a, NMDAR2b, NMDAR2c, NMDAR3a, and NMDAR3b were similar in both Tx3m and Naive groups, but the expression of AMPAR3 was significantly lower in the Tx3m group compared to the Naive group (Fig. 6a). The expression of postsynaptic scaffold molecules Homer1, Homer2, Homer3, Shank1, Shank2, Shank3, SAP97, PSD95, GKAP, Neuroligin1, Neuroligin2, Neuroligin3, N-cadherin, Neuregulin, and GRIP1 were comparable between the two groups (Fig. 7a). These results suggest that the decreased neuronal activity in the lumbar motor neurons after thoracic SCI is caused by the decreased presynaptic input from descending fibers and not the synaptogenic potential of the lumbar motor neurons.

### Motor neurons in the chronically injured spinal cord exhibit altered expression of factors involved in synapse formation

**Fig. 8:**
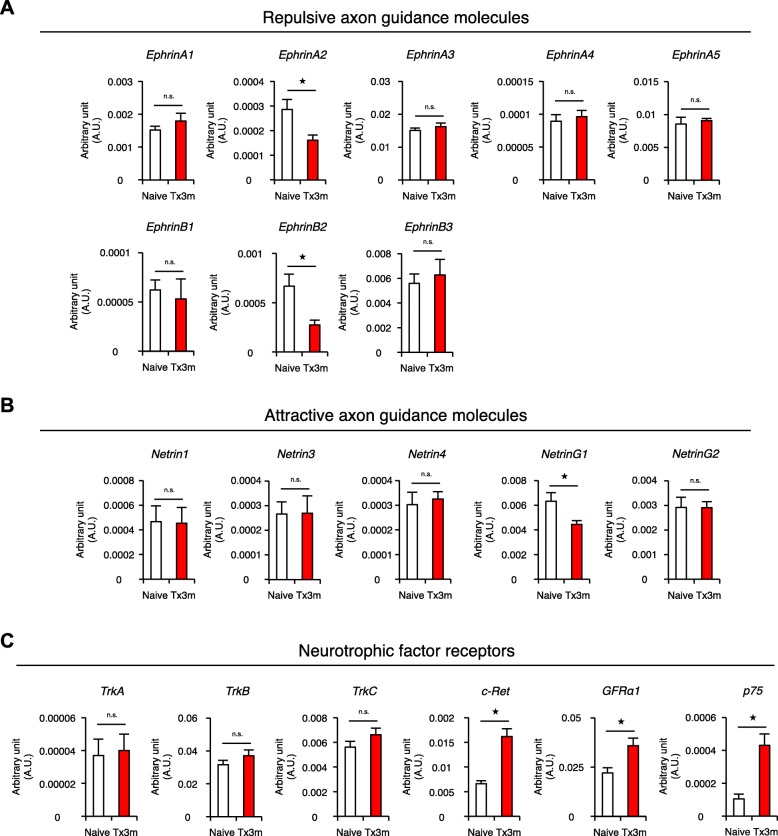
Expression of axon guidance molecules and neurotrophic factor receptors in the motor neurons changes in the chronic phase of SCI. mRNA expression of repulsive guidance molecules (**a**), attractive guidance molecules (**b**), and neurotrophic factor receptors (**c**) in lumbar motor neurons isolated by LMD in the Naïve and Tx3m groups (*n* = 8 mice per group). *P < 0.05, n.s. = not significant (*P* > 0.05), Wilcoxon rank sum test (**a**, **b**, and **c**). The data are presented as the mean ± SEM

Axon guidance molecules such as Ephrins (repulsive molecules) and Netrins (attractive molecules) have been shown to be key players in the formation of synapses [19, 20]. To examine the effect of SCI on these molecules, lumbar motor neurons in the chronic phase of SCI were collected by LMD and the expression of axon guidance molecules were examined by RT-PCR. Of the eight Ephrin ligands that have been identified in humans, the expressions of Ephrin-A2 and Ephrin-B2 were significantly decreased in the Tx3m group compared to the Naive group, while remaining Ephrin-A1, Ephrin-A3, EphrinA-4, Ephrin-A5, Ephrin-B1, and Ephrin-B3 were comparable between the two group groups (Fig. 8a). With regard to the attractive guidance molecules, Netrin-G1 expression was significantly lower in the Tx3m group compared to the Naive group, but there were no differences found in the expression of Netrin-1, Netrin-3, Netrin-4, and Netrin-G2 (Fig. 8b).

The expression of neurotrophic factor receptors were also examined because they have been shown to affect synapse formation [21]. In the isolated motor neurons, the gene expression of c-Ret (tyrosine kinase receptor), GFRa1 (GDNF family receptor a), and p75 (NGF receptor) were significantly increased in the Tx3m group compared to the Naive group, while the gene expression of TrkA (Tropomyosin-receptor-kinase A), TrkB, and TrkC were comparable between the two groups (Fig. 8c). Although the significance of these changes are not immediately apparent, changes in the expression of axon guidance molecules and neurotrophic factor receptors may reflect an altered potential for synaptogenesis in the lumbar motor neurons of the chronically injured spinal cord.

### Cholinergic activity is maintained in lumbar motor neurons in the chronic phase of SCI

**Fig. 9:**
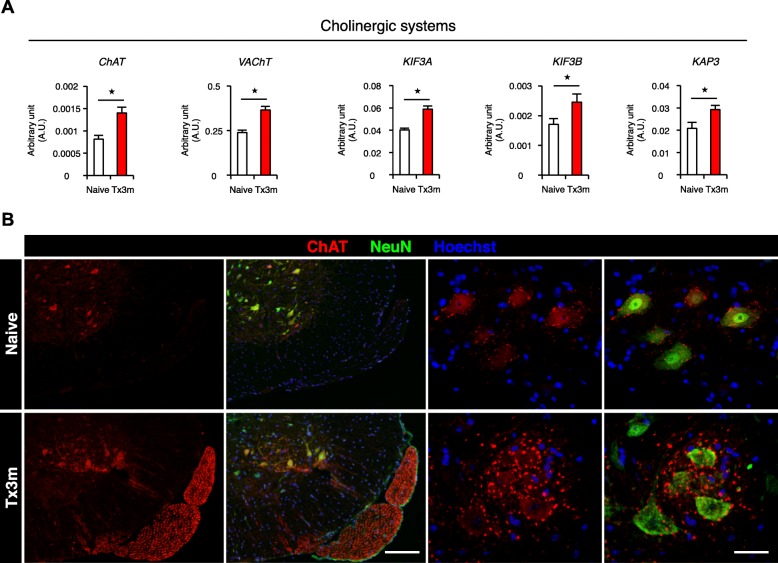
The expression of the acetylcholine-related molecules and their axonal transportation are upregulated in motor neurons in the chronic phase of SCI. **a** mRNA expression of acetylcholine-related molecules in lumbar motor neurons isolated by LMD in the Naive and Tx3m group (*n* = 8 mice per group). **b** Immunohistochemistry for choline acetyltransferase in the lumbar motor neurons stained with ChAT (red), NeuN (green), and Hoechst (blue). Significantly larger number of ChAT-positive boutons are observed in the Tx3m group compared to the Naive group. *P < 0.05, n.s. = not significant (*P* > 0.05), Wilcoxon rank sum test (**a**). Data are presented as the mean ± SEM. Scale bars: 100 μm (**b**, left two panels); 20 μm (**b**, right two panels)

Since a majority of lumbar motor neurons are cholinergic [22], we then examined the cholinergic activity of lumbar motor neurons. Even though neuronal activity markers were decreased in the lumbar motor neurons of the chronically injured spinal cord, the gene expression of ChAT (choline acetyltransferase), VAChT (choline vesicle transporter), and other choline-related transport molecules such as KIF3A, KIF3B, and KAP3 were increased in the Tx3m group compared to the Naive group (Fig. 9a). This did not reflect a general increase in axonal transport activity, because the gene expression of the other types of axonal transport molecules such as KIF1B, KIF5A, KIF5B, KIF5C, KIF13A, and KIF17 were comparable between the two groups (Additional file 3: Figure S3A). Consistent with the gene expression results, immunohistology confirmed the expression of ChAT in the lumbar motor neurons of the Tx3m group (Fig. 9b). These results suggest that the lumbar motor neurons selectively maintain cholinergic activity in the chronic phase of SCI even though synaptic input and general neuronal activity is depressed.

## Discussion

In this study, we reported three significant findings concerning the spinal cord in the chronic phase after thoracic SCI in comparison to a normal spinal cord. First, the size of the spinal cord decreases not only at the widely-known are near the injury epicenter, but also in a wide range extending rostrally and caudally. Second, the number of the presynaptic boutons and expression of neuronal activity markers are significantly lower in lumbar motor neurons, illustrating a decreased state of activity that occurs secondary to the decrease in descending synaptic input. Third, the lumbar motor neurons maintain the expression of postsynaptic molecules and cholinergic activity even though they are in a semi-dormant state. Notably, LMD method provided us with reliable data of cell-selective gene expressions in lumbar motor neurons regarding synaptogenic potential, axonal guidance molecules, neurotrophic factor receptors, and axonal transport molecules. The lumbar motor neurons maintained both in number and in size even in the chronic phase of SCI, and these neurons would not be dormant after SCI and possibly restore the neuronal activity by raising the synaptic input to distant caudal part from the lesion site.

Our results revealed a decrease in the white matter both rostral and caudal to the lesion. In the rostral spinal cord, there was a significant reduction of the dorsal column in the rostral spinal cord, which corresponds to the CST. The prominent decrease of the dorsal column area suggests that the retrograde dieback of the CST fibers demonstrated by Seif and colleagues [23] may be a major factor in the reduction of the rostral white matter area. There was a similar reduction of the dorsal column in the caudal spinal cord, although not as prominent, and we believe that the anterograde degeneration of axons (also known as Wallerian degeneration) is responsible for this change. When we examined the gray matter, there was a significant decrease in the area of the gray matter caudal to the lesion, but no change was detected in the rostral spinal cord. Since the number and size of the motor neurons were not affected by the spinal cord injury, we believe this difference is due to the degeneration of axonal fibers within the gray matter. Tracing experiments have demonstrated the presence of CST fibers within the gray matter [24–26], presumably from the dorsal column to the ventral horn where they form synapses with secondary motor neurons. The collective loss of CST fibers within the gray matter at all levels of the lumbar spinal cord due to anterograde degeneration may lead to a reduction of the gray matter caudal to the lesion. In the spinal cord rostral to the lesion, the CST fibers maintain connection with their targets and do not degenerate. Furthermore, since ascending sensory fibers transverse the gray matter at the level they enter the spinal cord and do not significantly cross into the gray matter on their path to the brainstem [27–29], the gray matter rostral to the lesion should not be largely affected.

Immunohistological analysis of the area distal to the injury confirmed that the number and size of lumbar motor neurons were unchanged even in the chronic phase. Although the number of presynaptic boutons in lumbar motor neurons were significantly decreased (Fig. 3), it is noteworthy that transection of the spinal cord did not lead to the complete loss of presynaptic molecules in the distal part of the lesion. In fact, we observed spatial connectivity between the presynaptic and postsynaptic molecules in lumbar motor neurons. These findings support the concept that the endogenous neuronal circuits remain intact within the distal spinal cord even though the circuits to the proximal neural pathways are severely disrupted [30]. Given that the synaptic transmission within the distal spinal cord is maintained [31], therapeutic interventions that bridge the impaired circuitry of the lesion could be a potential strategy to overcome the neural roadblock of the injury site.

Both vGluT2-positive and Bassoon-positive synaptic boutons decreased in the distal spinal cord after a transection injury. Considering that a recent report demonstrated that excitatory neurons directly contribute to motor function [32], the decrease in excitatory vGluT-positive synapses may be a potential intervention target to treat spinal cord injury. We did not specifically look into the changes that occur in the inhibitory synapses, but the decrease in Bassoon-positive synapses suggests a parallel decrease in inhibitory synapses [33, 34]. Although Bassoon has been used as a pan-synaptic marker that includes both excitatory and inhibitory synapses [35], Bassoon immunoreactivity encompasses most inhibitory synapses and gives it a more inhibitory profile.

The gene expression profile of the lumbar motor neurons from the Tx3m group revealed a significant decrease in the repulsive axon guidance molecules Ephrin-A2 and Ephrin-B2 and the attractive axon guidance molecule Netrin-G1, while the neurotrophic factors c-Ret, GFRα1, and p75 increased in the Tx3m group compared to the naive group. The decrease in repulsive axon guidance molecules and the increase in neurotrophic factors can be interpreted as the physiological reaction of lumbar motor neurons to the transection injury, attempting to promote axonal regeneration of descending tracts to their targeted lumbar motor neurons [36, 37]. The significance of the decreased Netrin-G1 is elusive, but suggests a potential therapeutic target to promote axonal regeneration in the injured spinal cord [38]. The role of axon guidance molecules has not been sufficiently studied in the injured spinal cord [39], and future studies are required to investigate their function during the regenerative process after spinal cord injury.

Rehabilitation programs such as physical therapy and occupational therapy are established treatment strategies for SCI patients [40, 41], that have been reported to be effective even for complete SCI patients [42]. Physiological exercise after SCI is reported to activate the reorganization of neuronal network in the distal part of the lesion [43], with passive locomotive training even inducing recovery of motor function in cats with complete SCI [44]. The effects of electrical stimulation are believed to induce functional recovery after SCI through a similar process [45], but the mechanisms underlying the restorative effect of rehabilitation for SCI remains elusive. Since our results demonstrate that the expressions of postsynaptic molecules are maintained in motor neurons distal to the lesion, rehabilitation program may promote synaptic reorganization of lumbar motor neurons that rewires the connection with proximal circuits. Our findings are in line with other studies that strongly suggest that the stimulation of muscles and peripheral nerves could be the catalyst for a reorganization of the neural network to the distal spinal cord.

Contrary to our expectation, the synaptogenic potential of lumbar motor neurons were maintained in the chronic phase of SCI. This suggests that if a means to transmit neural signals across the SCI lesion site was established, the conduction of proximal signals from the brain to peripheral systems may be reestablished. Indeed, Lu and colleagues demonstrated that the neural stem cells transplanted into the injured spinal cord formed synaptic connections with lumbar motor neurons distal to the lesion [46]. This may also imply that we should reconsider the ideal location to transplant neural stem cells into the injured spinal cord; the lesion epicenter may not necessarily be the ideal place for stem cell transplantation when considering the synaptic connectivity with the motor neurons of the distal spinal cord.

In this study, LMD was an indispensable tool to analyze cell-selective gene expression in lumbar motor neurons. By reliably collecting only motor neurons, we were able to obtain critical information pertaining to the synaptogenic factors, axonal guidance molecules, neurotrophic factor receptors, and axonal transport molecules of the lumbar motor neurons. Previously, fluorescence-activated cell sorting (FACS) has thus far been the prevailing method for cell-specific analyses [47]. However, LMD procedure provides certain advantages over FACS. First, LMD enables the collection of neuronal cells without disrupting their distinctive structures, such as long axons and dendrites. This has tremendous value, because the majority of synaptic mRNAs are localized throughout the axonal and dendritic terminals which are hundreds of micrometers distant from the neuronal cell body [48]. Furthermore, FACS requires enzymatic dissociation to isolate neuronal cells from spinal cords and eliminate myelin and extracellular matrix protein, but this enzymatic dissociation often results in a loss of cellular mRNA and proteins [49]. We acknowledge the advantage of FACS in analyzing blood cells and culture cells, but in the analysis of cells integrated into a cellular matrix or a neuronal network, the ability to analyze the gene expression of cells in their original environment and morphology with intact cellular connections is a tremendously useful tool, especially for the investigation of neuronal cells in the CNS.

In conclusion, we clarified the pathologic changes of the lumbar spinal cord in the chronic phase of complete thoracic SCI. The lumbar motor neurons remote to the lesion received less synaptic input and exhibited lower neuronal activity. However, our findings reveal that these motor neurons are not dormant and persistently express postsynaptic molecules even in the chronic phase of SCI. Indeed, the cholinergic potential of lumbar motor neurons were even higher than in naive animals, suggesting that these neurons are primed and ready for any input that comes their way. Our findings suggest that the neural pathways distal to the SCI lesion may be relatively intact, and that what is desperately desired is a dependable means to restore synaptic input to the lumbar motor neurons.

## Methods

### Surgical procedures

Adult female C57/BL6 mice were anesthetized via an intraperitoneal injection of pentobarbital (75 mg/kg) and received a complete spinal cord transection at the 9th thoracic level (*n* = 30). Through a longitudinal incision, the paraspinal muscles were sharply dissected and a T9 laminectomy was performed to expose the spinal cord. The spinal cord was completely transected at the T9 level with microscissors (including the dura mater). The cut ends of the spinal cord were lifted with small forceps to assure complete transection. The muscles and skin were subsequently sutured in layers. All animal studies were conducted in accordance with the institutional guidelines and regulations for animal experiments and were approved by the Committee of Ethics on Animal Experiment in Faculty of Medicine, Kyushu University.

### Histopathological examination

After the mice were transcardially fixed with 4% paraformaldehyde, the spinal cord was removed, dehydrated and embedded in OCT compound. The frozen tissue was cut in the sagittal or axial plane in 16 μm sections. For immunostaining, the sections were stained overnight at 4 °C with primary antibodies against MBP (myelin marker, 1:200, rat, Chemicon), NF200 (neuronal fiber marker, 1:200, rabbit, Sigma-Aldrich), NF200 (1:200, mouse, Sigma-Aldrich), 5-HT (serotonergic marker, 1:200, goat, ImmunoStar), VGluT2 (presynaptic marker, 1:200, guinea pig, Synaptic Systems), PSD95 (postsynaptic marker, 1:200, rabbit, Invitrogen), Hu (neuronal marker, 1:1000, human, a gift from Dr. Robert Darnell, The Rockefeller University, New York, NY, USA), Bassoon (pan-presynaptic marker, 1:200, mouse, Stressgen), NeuN (neuronal marker, 1:1000, mouse, Chemicon), c-fos (neuronal activity marker, 1:200, rabbit, Santa Cruz Biotechnology), or ChAT (1:100, goat, Chemicon). Then the sections were then incubated at room temperature for 1 h with Alexa Fluor-conjugated secondary antibodies (1:200, Invitrogen). Nuclear counterstaining was obtained using Hoechst 33342 (Molecular Probes).

### Laser-capture microdissection

The injured spinal cords were resected at 3 months after injury, immediately frozen in dry ice/hexane, and stored at − 80 °C. The tissues were sectioned into 16 μm thick slices using a cryostat at − 20 °C and were mounted on PEN membrane slides. The sections were then fixed in ice-cold acetone for 2 min. Nissl-stained motor neurons in the ventral horn that were over 20 μm in diameter were selected based on morphological criteria [50, 51] and dissected with the LMD 6500 system (Leica Microsystems) into a microcentrifuge tube cap placed directly beneath the section. The tube cap was filled with 75 μl of RLT buffer (Qiagen). For each sample, 500 cells were dissected from one series of axial sections.

### Quantitative RT-PCR

Total RNA was isolated from the spinal cord using an RNeasy Micro Kit (Qiagen). For complementary DNA (cDNA) synthesis, the reverse transcriptase reaction was performed using a Prime Script first-strand cDNA Synthesis Kit (Takara Bio). Quantitative RT-PCR was performed using primers specific to the genes of interest (Additional file 4: Table S1) and SYBR Premix Dimmer Eraser (Takara Bio). The data were normalized to glyceraldehyde-3-phosphate dehydrogenase. RT-PCR was conducted using a Thermocycler (Biometra, Göttingen, Germany) and products were detected by electrophoresis and ethidium bromide staining.

### Image acquisition and quantitative analysis

All images were obtained using a LSM510 laser scanning microscope system (Zeiss) or a BZ-9000 digital microscope system (Keyence). To quantify the number of neurons, we performed immunostaining with an anti-Hu (neuronal marker) antibody in serial sections and counted the number of immunoreactive cells that were present 6 mm distant from the lesion epicenter. The motor neurons (cell diameter > 20 μm) in the ventral horn were selected under the microscope based on morphological criteria [50]. For quantification of the presynaptic boutons (Bassoon and VGluT2) in lumbar motor neurons, immunostaining of sagittal sections was performed using each antibody. The algorithms for counting the number of synaptic boutons were performed as described previously [52, 53]. Briefly, 40 optical slices of axial sections at × 1000 magnification were acquired 0.4 μm intervals and reconstructed into a single image, and immunopositive synaptic boutons in each motor neuron were counted. Two motor neurons were analyzed from each mouse (8 neurons from 4 mice in total) and the total number of synaptic boutons observed in each motor neuron was counted and presented as a bar graph. Only neurons with a nucleus and proximal neurite processes that were confirmed by focusing up and down through the thickness of the section were included in the cell counts.

### Statistical analysis

The Wilcoxon rank sum test was used to compare the medians of the data for quantitative RT-PCR as well as the area of spinal cord, the area of immunopositive area, and the number of synaptic boutons. In all statistical analyses, significance was defined as *P* < 0.05. The values for groups are presented as the average ± SEM. All statistical analyses were carried out using the JMP software program (version 13; SAS Institute).

## Additional files

Additional file 1: **Figure S1.** Large Hu-positive neurons located in ventral horn of lumbar spinal cord are ChAT-positive motor neurons. **A:** Immunohistochemistry for the motor neuron-specific marker ChAT (red) and the neuronal marker Hu (green) in the lumbar spinal cord, with nuclear counterstain in Hoechst (blue). The large neurons located in the ventral horn of the lumbar spinal cord are immunopositive for both ChAT and Hu. (PDF 497 kb) Scale bars: 100 μm (**A**). (PDF 27 kb)

Additional file 2: **Figure S2.** Lumbar motor neurons in the distal spinal cord are not lost after SCI. Both the area (A) and the number (B) of lumbar motor neurons are comparable between the Naive group and Tx3m groups (*n* = 8 mice per group). *P < 0.05, n.s. = not significant (*P* > 0.05), Wilcoxon rank sum test (**A**). Data are presented as the mean ± SEM. (PDF 27 kb)

Additional file 3: **Figure S3.** Expression of axonal transport molecules are maintained in lumbar motor neurons in the chronic phase of SCI. (PDF 32 kb). **A:** mRNA expression of kinesin superfamily proteins in lumbar motor neurons isolated by LMD in the Naive and Tx3m groups (*n* = 8 mice per group). (DOCX 27 kb) *P < 0.05, n.s. = not significant (*P* > 0.05), Wilcoxon rank sum test (**A**). Data are presented as the mean ± SEM.0.

Additional file 4: **Table S1.** Primers used for quantitative RT-PCR (DOCX 27 kb)

## Abbreviations

5HT: 5-Hydroxytryptamine
AMPAR: α-amino-3-hydroxy-5-methyl-4-isoxazole propanoic acid receptor
CACNG2: Calcium channel voltage-gated channel auxiliary subunit gamma-2
CaMK2a: Ca2+/calmodulin-dependent protein kinase-2a
ChAT: Choline acetyltransferase
CNS: Central nervous system
FACS: Fluorescence-activated cell sorting
GKAP: Guanylate kinase-associated protein
GRIP1: Glutamate receptor-interacting protein-1
KAP3: KIF-associated protein-3
KIF: Kinesin superfamily protein
LMD: Laser microdissection
MBP: Myelin basic protein
NF200: Neurofilament-200
NMDAR: N-methyl-D-aspartate receptor
PSD95: Postsynaptic density protein-95
SAP97: Synapse associated protein-97
SCI: Spinal cord injury
VAChT: Vesicular acetylcholine transporter
vGluT2: Vesicular glutamate transporters-2
