# Supplementary figures and images for: Pathological changes of distal motor neurons after complete spinal cord injury
Source: Mol Brain. 2019 Jan 9;12:4. doi: 10.1186/s13041-018-0422-3 (PMC6327522; doi:10.1186/s13041-018-0422-3)

A

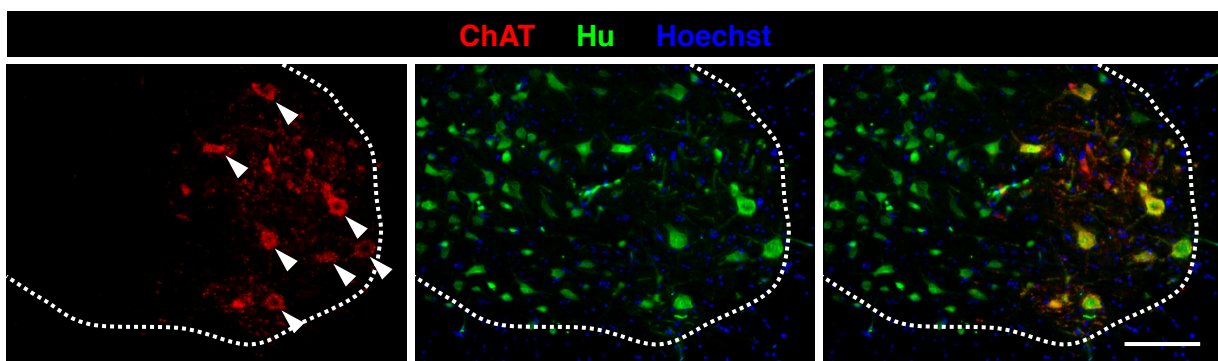

Supplemental figure 1

Supplement: Supplementary file 1 — Figure S1. Large Hu-positive neurons located in ventral horn of lumbar spinal cord are ChAT-positive motor neurons. A: Immunohistochemistry for the motor neuron-specific marker ChAT (red) and the neuronal marker Hu (green) in the lumbar spinal cord, with nuclear counterstain in Hoechst (blue). The large neurons located in the ventral horn of the lumbar spinal cord are immunopositive for both ChAT and Hu. (PDF 497 kb) Scale bars: 100 μm (A). (PDF 27 kb) [file 13041_2018_422_MOESM1_ESM.pdf]

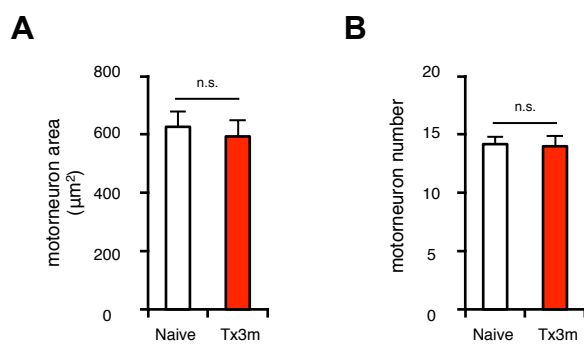

Supplemental figure 2

Supplement: Supplementary file 2 — Figure S2. Lumbar motor neurons in the distal spinal cord are not lost after SCI. Both the area (A) and the number (B) of lumbar motor neurons are comparable between the Naive group and Tx3m groups (n = 8 mice per group). *P < 0.05, n.s. = not significant (P > 0.05), Wilcoxon rank sum test (A). Data are presented as the mean ± SEM. (PDF 27 kb) [file 13041_2018_422_MOESM2_ESM.pdf]

**A** Kinesin superfamily proteins (KIFs)

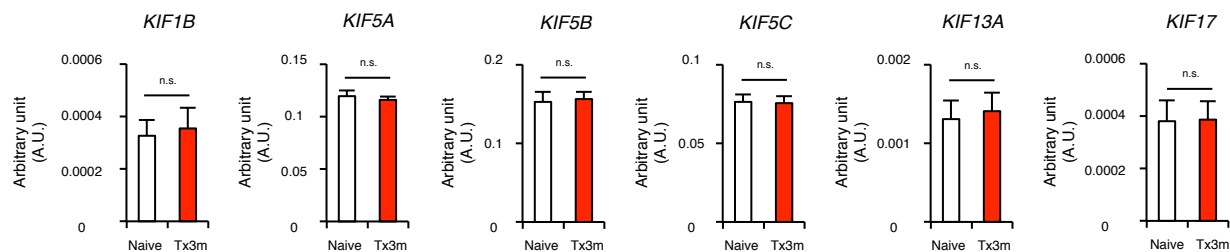

Supplement: Supplementary file 3 — Figure S3. Expression of axonal transport molecules are maintained in lumbar motor neurons in the chronic phase of SCI. (PDF 32 kb). A: mRNA expression of kinesin superfamily proteins in lumbar motor neurons isolated by LMD in the Naive and Tx3m groups (n = 8 mice per group). (DOCX 27 kb) *P < 0.05, n.s. = not significant (P > 0.05), Wilcoxon rank sum test (A). Data are presented as the mean ± SEM.0. [file 13041_2018_422_MOESM3_ESM.pdf]
