# Supplementary material for: Pathological changes of distal motor neurons after complete spinal cord injury
Source: Mol Brain. 2019 Jan 9;12:4. doi: 10.1186/s13041-018-0422-3 (PMC6327522; doi:10.1186/s13041-018-0422-3)
Supplement: Supplementary file 4 — Table S1. Primers used for quantitative RT-PCR (DOCX 27 kb) [file 13041_2018_422_MOESM4_ESM.docx]

**Table S1. Primers used for quantitative RT-PCR**

| Gene symbol | Accession number | 5’- Forward primer -3’ | 5’- Reverse primer -3’ |
| --- | --- | --- | --- |
| *GAPDH* | NM_008084.3 | gacttcaacagcaactcccactct | ggtttcttactccttggaggccat |
| *ChAT* | NM_009891.2 | tatctatctaagtggcctgtggcc | tgagatgacgattcttccactggc |
| *CNPase* | NM_001146318.1 | aaatggcagaccagtatcagtacc | gtctcagaactctttttggtcagg |
| *GFAP* | NM_001131020.1 | tgtactaacagagcgagcctatgc | gggacttgctgcctttaacattgg |
| *C-fos* | NM_010234.2 | acctactgtgttcctggcaatagc | tgtcagaacattcagaccacctcg |
| *CaMK2a* | NM_009792.3 | gaggagtgagaggaagttttaacc | tgactcactgtgtgttagtcatgc |
| *AMPAR1* | NM_001113325.2 | ttctgtgctgtttcctgtcctagg | catcttaaggtcccctgatgtacc |
| *AMPAR2* | NM_001083806.1 | tatggcaggctttggtttggttcc | accatttccttagtctgtgtgcgg |
| *AMPAR3* | NM_001281929.1 | tgtttggagaactccccatagtgg | tatctgtgggctgtaattccaggc |
| *AMPAR4* | NM_019691.4 | gtttagttttttggggggagtggg | acacaggtagcaggttctttgacc |
| *Stargazin* | NM_007583.2 | cccgcattcttttccctttaaccc | attggtgctggatagagttggtcg |
| *NMDAR1* | NM_008169.3 | cctcacttttgagaacatggcagg | gtgatatcagtgggatggtactgc |
| *NMDAR2a* | NM_008170.2 | acccccagggttttaaattcctgc | ttcctcctagtagggtgctattgg |
| *NMDAR2b* | NM_008171.3 | agagagattcctgaacgaagtggg | aagcgcattgctttcgtatgtggg |
| *NMDAR2c* | NM_010350.2 | atcttccatggccagcttatgacc | cctccgtttccaattttgtgtccc |
| *NMDAR2d* | NM_008172.2 | ctaagtggacaagaccctcatggg | cgggtttaaaaaagagtcggtggg |
| *NMDAR3a* | NM_001276355.1 | ttcactgaccaccaatgggaaagc | gatactcctcaagctctgtcttcc |
| *NMDAR3b* | NM_130455.2 | ttctgagcaacacctcatttcagg | aatgggtgttccaccagagttacc |
| *Homer1* | NM_0011982.3 | actgtttatggactgggattctcc | gtctgtcccattgatactttctgg |
| *Homer2* | NM_0011983.2 | agacatcgaggagagtaaataccg | aatggagaatgtcctacgtctacc |
| *Homer3* | NM_001146153.1 | taccgtgtcctatttctatgatgc | acttctgagaggttttggtgaagg |
| *Shank1* | NM_001034115.1 | tactgcaccctttctttctctagc | gttcagagcatgtacagagagagc |
| *Shank2* | NM_001081370.2 | cccacactctcagatgtctttagc | gtggacaggcttagttgtaaaagg |
| *Shank3* | NM_021423.3 | attccttagacacagagtcacacc | ccagatggaactccatacaaaagg |
| *SAP97* | NM_007862.3 | cattaaaggtcctaaaggtcttgg | ttctaaacacacactgttcactgc |
| *PSD95* | NM_001109752.1 | ctacgtaaccaagatcatcgaagg | acaacgtcatatgtgttcttcagg |
| *GKAP* | NM_177639.6 | gttggttctgtcagtttagtgtcc | gaaatgtgaactagacaggtgtgg |
| *Neuroligin1* | NM_138666.3 | ttactctgaagtttgctcctttcg | gagtaaattcaagagaccctcagc |
| *Neuroligin2* | NM_198862.2 | gagtcagggaaaaaggttacatgg | aatacatgaagattcagccacacc |
| *Neuroligin3* | NM_172932.4 | catacaatcagaccaaggagaagg | tgtaggctctgtagtcctctctgg |
| *N-cadherin* | NM_007664.5 | catctataatgctaccttccttgc | tgggtctatgtcataatcaagtgc |
| *Neuregulin* | NM_178591.2 | ctcgtatgtcacctgtagatttcc | gggtgatgatcatacttcttctcc |
| *GRIP1* | NM_028736.2 | ggtgaccttatacaaggactctgg | ttacctgtaagagcctgtcgtagg |
| *EphrinA1* | NM_010107.4 | gcctcaaaaggttcagtattaagg | cagcttgtttctttggcttaaagg |
| *EphrinA2* | NM_007909.3 | gggtggactttttaggatagaagc | cctgctgtatttttggtaggtagc |
| *EphrinA3* | NM_010108.1 | ggtctgcactgtacatctctctgg | agtcctgagcactgcctttatagc |
| *EphrinA4* | NM_007910.2 | agacaggatgaaactttgatgtgc | atcaaagcagctatgctatgttcc |
| *EphrinA5* | NM_207654.2 | aactgcaccctagagagttgtacg | tctgtgatgagactctgctactcc |
| *EphrinB1* | NM_010110.5 | ctccttagtagttcaggggagtgg | cattaattaccgtgagagtgaagg |
| *EphrinB2* | NM_010111.5 | gtagttctttccatcaccctaacc | tcatccgattcttcacagtttagc |
| *EphrinB3* | NM_007911.5 | cctgcctttctgtgtacttactcc | aacgagtcacttcctatctgatgc |
| *Netrin1* | NM_008744.2 | gaacaagactgaaatgaggagacc | cacaggaatatctgtctgtgaacc |
| *Netrin3* | NM_010947.3 | gcttcaggagttttgaataacagc | ctttaaagttagcccagtgagtcc |
| *Netrin4* | NM_021320.3 | ttgcacgtctagatactcaaatcc | gttcaggtcgtttatctgacaagg |
| *NetrinG1* | NM_001163348.1 | ccacttcacaagagtggttagagg | actcaaacatcagtcagtctttgc |
| *NetrinG2* | NM_133500.2 | ttatgtttgacagggaagatgagg | gtcctctgcatagaactggtaggg |
| *TrkA* | NM_001033124.1 | gtttcatcttcactcagttcttgg | ctcaaaagggttgtccataaaagc |
| *TrkB* | NM_008745.3 | ctctcacctgtcttgtaactgtgc | agctactgatctggtctcctttcc |
| *TrkC* | NM_008746.5 | acatcgtgttgaagagagaattgg | accccatagaacttgacaatatgc |
| *c-Ret* | NM_001080780.1 | agcctctgctaaattacctgtacc | ctgaagaactcaaacttcatgtgc |
| *GFRa1* | NM_010279.3 | ctccacagagtgtttatgtgatcc | ctacattcagcttctcactcaagc |
| *P75* | NM_033217.3 | agaggattacaggcctatctgagc | agatgtttccctgaaagtcactcc |
| *VAChT* | NM_021712.2 | caaacacactcgtattcattgagg | gtagtagaccaagtttggcactgg |
| *KIF3A* | NM_008443.4 | caactttacactcatcctgactgc | gagccaacttagtaacaacaatgg |
| *KIF3B* | NM_008444.4 | ctcagagggatctaggtaagaacg | atctaaagacagccaaagagaagg |
| *KAP3* | NM_010629.3 | gtgtgctttaccatataagcatgg | gagctggacatttctcttgttagc |
| *KIF1B* | NM_008441.2 | ggtctgaatgtattcttggtaggc | ctgtaacaaagtgagcctctatgg |
| *KIF5A* | NM_001039000.4 | cagatatgtactttgccagcagcg | gaaagtgcagatatgtggccaggg |
| *KIF5B* | NM_008448.3 | taccctgtacagctgtttcctacc | agtccctcaacacagttactcagc |
| *KIF5C* | NM_008449.2 | gaatttgtgtgcacagatacgtgc | acttgattgtccctctaggatggc |
| *KIF13A* | NM_010617.2 | gagaagctgataaaggagctaacg | tagtaaaccagaagctcgttgagg |
| *KIF17* | NM_001190978.1 | cttccctattccctagagaactcc | cttctgtgctctgtatgagaatgc |
